# Panel-Based Population Next-Generation Sequencing for Inherited Retinal Degenerations

**DOI:** 10.1038/srep33248

**Published:** 2016-09-14

**Authors:** Matthew Carrigan, Emma Duignan, Conor P. G. Malone, Kirk Stephenson, Tahira Saad, Ciara McDermott, Andrew Green, David Keegan, Peter Humphries, Paul F. Kenna, G. Jane Farrar

**Affiliations:** 1 School of Genetics and Microbiology, Trinity College Dublin, Dublin, Ireland; 2 Research Foundation, Royal Victoria Eye and Ear Hospital, Dublin, Ireland; 3 Mater Misericordiae University Hospital, Dublin, Ireland; 4 Our Lady’s Children’s Hospital, Crumlin, Dublin, Ireland

## Abstract

Inherited retinopathies affect approximately two and a half million people globally, yet the majority of affected patients lack clear genetic diagnoses given the diverse range of genes and mutations implicated in these conditions. We present results from a next-generation sequencing study of a large inherited retinal disease patient population, with the goal of providing clear and actionable genetic diagnoses. Targeted sequencing was performed on 539 individuals from 309 inherited retinal disease pedigrees. Causative mutations were identified in the majority (57%, 176/309) of pedigrees. We report the association of many previously unreported variants with retinal disease, as well as new disease phenotypes associated with known genes, including the first association of the *SLC24A1* gene with retinitis pigmentosa. Population statistics reporting the genes most commonly implicated in retinal disease in the cohort are presented, as are some diagnostic conundrums that can arise during such studies. Inherited retinal diseases represent an exemplar group of disorders for the application of panel-based next-generation sequencing as an effective tool for detection of causative mutations.

Inherited retinal disorders (IRDs) comprise a broad spectrum of genetically heterogeneous conditions with overlapping clinical presentations. The most common diagnosis in this disease spectrum is retinitis pigmentosa (RP), with a worldwide prevalence of approximately 1/3000^1^. RP is one of the most genetically diverse Mendelian diseases, with mutations in over 60 genes currently implicated as causative of disease, and is marked by a progressive loss of photoreceptor cells, typically involving a rod-cone degeneration with an associated loss of visual function^2^. Mutations in over 200 genes have been implicated in inherited retinal disorders as a whole to date^3^, with the result that traditional sequencing approaches are usually inadequate for the task of identifying causative mutations.

The arrival of next-generation sequencing (NGS) technologies offers for the first time an opportunity to locate these mutations. NGS technology has advanced at an exponential pace, with the cost per megabase of sequence halving regularly^4^,^5^. This has enabled enhanced diagnosis for both genetic and infectious diseases^6^, as well as providing insights into mutations driving tumorigenesis. Few conditions, however, have seen the same level of change as IRDs, where NGS has almost completely supplanted previous approaches.

We present results from the *Target 5000* project, a NGS study aimed at identifying causative mutations in an extensive Irish IRD patient cohort. To date, probands from 309 IRD pedigrees have been sequenced. DNA samples were sequenced using a capture panel consisting of all coding exons for genes previously implicated in retinopathies as listed by Retnet^3^ (Supplementary Table 1). The data from prior studies have suggested that over fifty percent of disease-causing mutations will fall in the exons of known retinopathy genes^7^, and hence targeting these exons allows rapid and cost-effective identification of causative mutations for the majority of patients, with improved sensitivity for mutations in the target region compared to whole-exome capture^8^.

Employing target-panel capture and sequencing and ensemble prediction methods for novel variant classification, candidate mutations were identified in 57% of pedigrees, despite the substantial genetic heterogeneity of these conditions. Many novel and previously reported mutations were identified in this IRD cohort, as were new ocular disease phenotypes associated with genes previously implicated in other retinal disorders.

## Results

### Sample Acquisition and Sequencing

**Figure 1:**
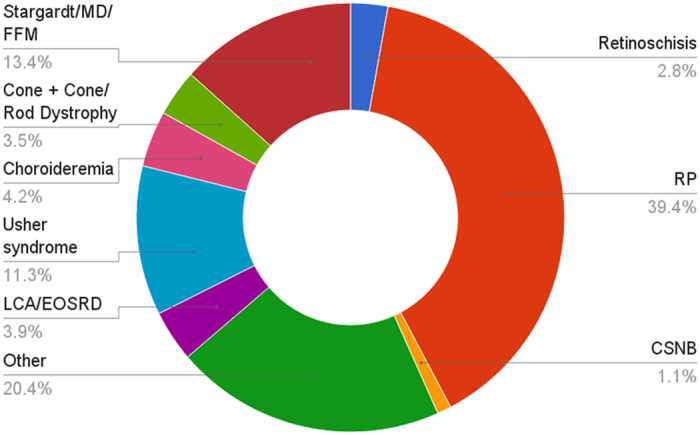
Breakdown of patient cohort by condition.

Probands from a total of 309 Irish IRD pedigrees were sequenced. Following informed consent, samples were taken and clinical examinations performed. Patients over 18 years with an IRD were eligible to enrol in the study. A full breakdown of the frequency of different conditions is provided in Fig. 1.

DNA was isolated and samples were prepared, target-captured and sequenced as described in the Methods section. The list of genes sequenced is given as Supplementary Table 1. Overall sequence quality was high. An average of 98.9**%** of the targeted coding sequence in each sample had 8X coverage or better (interquartile range 98.6–99.5%), with 95% covered at 30X coverage or better.

**Figure 2:**
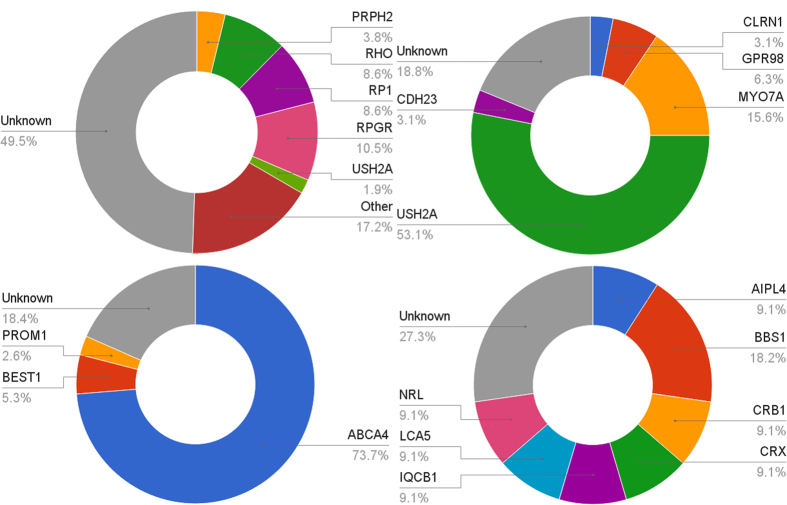
Prevalence of causative mutations in specific genes in patients with an initial diagnosis of: Top-left: Retinitis pigmentosa. Top-right: Usher Syndrome. Bottom-left: Stargardt Disease or macular dystrophy. Bottom-right: Leber Congenital Amaurosis or Early-Onset Severe Retinal Degeneration.

Overall, causative mutation(s) could be identified in 57% (176/309) of sequenced pedigrees, although this proportion differed substantially between different conditions (see Fig. 2). A substantial number of novel mutations that had not previously been associated with retinal disease were identified (Table 1).

### Stargardt Disease

The most commonly implicated gene in Stargardt disease was *ABCA4*, causing recessive Stargardt disease or Fundus Flavimaculatus in 32 pedigrees. Although Stargardt disease is not the most common condition in the Irish IRD patient cohort, it is very genetically homogeneous: 73% of sequenced Irish Stargardt pedigrees showed causative mutations in *ABCA4,* with many of the remainder having one identifiable *ABCA4* mutation but not two. *ABCA4* mutations are the primary cause of Stargardt disease and autosomal-recessive cone-rod dystrophy, in addition to causing a small number of cases of RP^9^; *ABCA4* was therefore the single most commonly implicated gene across all conditions in this study. This cohort included one pedigree with a rare case of dominant Stargardt-like disease caused by a mutation in the *PROM1* gene, (NM_006017.2:c.1117C > T,p.Arg373Cys), which has previously been identified as causative of dominant maculopathy^10^. In addition, mutations in *BEST1* were identified in one pedigree in the cohort, leading to a rediagnosis of Best Vitelliform Macular Dystrophy.

### Leber Congenital Amaurosis (LCA) and Early-Onset Severe Retinal Degeneration (EOSRD)

The cohort of IRD patients with LCA/EOSRD was relatively small, comprising 18 patients across 11 pedigrees. The mutation NM_001122769.2:c.1756A > T,p.Lys586* in the *LCA5* gene, which encodes the Lebercillin protein, segregated with the disease in a recessive LCA pedigree with two affected members. This mutation has not previously been reported as disease causing, although the variant was reported by the Exome Aggregation Consortium (ExAC)^11^ at an allele frequency of 1/121,012. No homozygotes have previously been reported. As a premature stop mutation in a gene implicated in LCA and showing segregation with disease, it is highly likely to be the causative mutation. The homozygous mutation (NM_000554.4:c.206G > A,p.Arg69His) in the *CRX* gene was observed in a simplex pedigree with no other candidate mutations. The Arg69His substitution has not been implicated in disease before, but has been observed in ExAC at extremely low allele frequency (2/121,402), with no homozygotes reported. The mutation is predicted by the ensemble model to cause disease and is located in the crucial homeodomain of CRX, a domain previously reported to be sensitive to missense mutations^12^. Sanger sequencing of the patient’s mother, who retains normal visual function, confirmed the heterozygous presence of the mutation, indicating that the mode of inheritance is indeed recessive.

In one interesting simplex case compound heterozygous frameshifts in the *NRL* gene, (NM_006177.3:c.16delA,p.Ser6fs) and (NM_006177.3:c.386delC,p.Ala129fs), were found in a patient with atypical LCA marked by unusually good retention of visual acuity despite the presence of typical LCA features such as congenital nystagmus, extinguished ERG and onset of visual symptoms at a very young age. Dominant-acting *NRL* mutations have been reported in the past^13^, and so molecular cloning of the entire region was used to verify that the two mutations were on separate chromosomes and did not merely represent a single haplotype. Mutations in *NRL* have also been implicated previously in recessive inherited retinal disease^14^, however we have been unable to find another reported case of a patient with two frameshift or nonsense mutations in *NRL*. As such, this is a phenotype of interest, as the first homozygous *NRL*-null case reported in humans.

At 46 years the proband retained best-corrected Snellen visual acuity of 6/60 and 6/15 in the right and left eyes respectively. Visual fields were concentrically constricted to within 10° of fixation to the Goldmann IV4e target. No convincing rod or cone full-field ERG responses were recordable, although delayed and reduced amplitude cone responses were recordable 10 years previously. Fundoscopy at 46 years of age revealed extensive retinal and choroidal atrophy peripherally, with better preservation at the posterior pole in each eye. Scattered clumped pigment deposits were observed, consistent with existing observations of patients with recessive *NRL*-based disease^14^.

### Retinitis Pigmentosa (RP)

The largest disease cohort comprised patients with RP, accounting for 37.9% of the total patient cohort. The most commonly implicated genes for RP varied based on inheritance: for dominant RP, the most commonly implicated genes were *RHO* (8.6%), *RP1* (8.6%) and *PRPH2* (3.8%). This may underestimate the true prevalence of *RHO* mutations in autosomal dominant RP, as pre-NGS sequencing studies had been performed in the Irish IRD population focusing solely on exons of *RHO*^15^,^16^. As a result, a number of patients with *RHO* mutations had already been identified, and this subpopulation of Irish IRD patients did not participate in the current study, resulting in a likely underestimate of the frequency of *RHO*-linked adRP.

The primary contributor to the high prevalence of RP1 mutations in dominant RP was a frameshift mutation in *RP1* (NM_006269.1:c.2285_2289delTAAAT,p.Leu762fs) that was observed to segregate with disease in five dominant RP pedigrees, including one large pedigree with four affected members and several unaffected members, and was never observed in pedigrees with other conditions. Although these patients presented to the clinic as unrelated probands and were not geographically clustered, it is anticipated that they likely share a common ancestor and form a contiguous super-pedigree. This *RP1* mutation has not been previously reported in dbSNP or ExAC, nor was it observed in a recent study of RP patients in Northern Ireland^17^ but is the most frequent single mutation causing RP in the current study.

Similarly, the primary contributor to the high prevalence of *PRPH2* mutations is the S212G (NM_000322.4:c.634A > G,p.Ser212Gly) mutation which has previously been identified as causative of disease in the Irish population^18^. As with the *RP1* p.Leu762fs mutation, patients carrying the S212G mutation likely share a common ancestor, although they presented as unrelated probands in the clinic.

**Figure 3:**
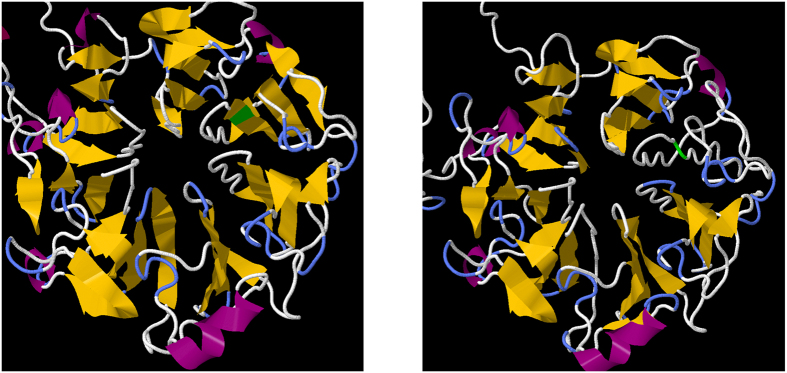
Left: A simulated fold of the beta-propeller domain in the native RPGR sequence shows the expected seven beta sheet propellers forming correctly. Thr99 is highlighted in green. Right: A simulated fold of the same domain in RPGR-T99A. Ala99 is highlighted in green. The entire propeller containing residue 99 has converted from sheet to coil conformation.

In cases of X-linked RP, nearly half of all sequenced pedigrees (10/21) showed mutations in the *RPGR* gene, representing almost all of the XLRP pedigrees for which mutations could be identified in this study, with the exception of two pedigrees with *RP2* mutations. The *RPGR* mutations included the previously-reported G436D (NM_001034853.1:c.1307G > A,p.Gly436Asp) mutation. However, most mutations in *RPGR* were premature stop and frameshift mutations. Several of these were novel mutations that have neither been reported previously nor implicated in disease (Table 1). There was also one novel amino acid substitution in the *RPGR* gene (p.Thr99Ala) that segregated with disease in a small pedigree. This region of the protein is highly conserved and quite sensitive to mutation^19^; a different mutation in the same codon (p.Thr99Asn)^19^, as well as a mutation in the adjacent codon (p.His98Gln)^20^ have been implicated in disease. Simulated folding of both the native and the novel p.Thr99Ala proteins using the *I-TASSER* suite^21^ showed a disruption in the beta-propeller domain that contains residue Thr99, converting that domain from beta-sheet to coil conformation. Such a conformational change would be likely to severely affect protein function (Fig. 3).

For recessive and simplex forms of RP, the results obtained were more disparate. Notably, however, four pedigrees within the simplex/recessive RP cohort were found to carry the *BBS1* mutation NM_024649.4:c.1169T > G,p.Met390Arg, which has been associated with Bardet-Biedl syndrome. In one of these cases, the patient was noted to have mild mental retardation consistent with Bardet-Biedl syndrome. However, the other three patients showed none of the extraretinal features of the disease. These cases therefore continued to be classified as nonsyndromic RP. This is consistent with reports from other groups, which have identified that *BBS1* p.Met390Arg can cause either Bardet-Biedl syndrome or nonsyndromic RP in different pedigrees^22^.

Two cases in the recessive/simplex RP cohort were particularly noteworthy: A homozygous premature stop mutation in the *GNAT1* gene (NM_000172.3:c.904C > T,p.Gln302*) in a case of simplex RP, which has been reported in an earlier paper^23^ and a homozygous frameshift in the *SLC24A1* gene (NM_004727.2:c.2679delT,p.Asn893fs) in a pedigree with two affected individuals. In both situations, the mutation segregated with the retinopathy (although in the case of the *GNAT1* mutation, there was only one patient who carried it homozygously and only this individual was affected in the pedigree). Both of these genes have been implicated in congenital stationary night-blindness (CSNB), but neither gene has previously been implicated in RP. Although both CSNB and RP affect rod photoreceptor cells, rod cells do not die in significant numbers in CSNB, with the result that the disease is largely stationary and does not affect central or colour vision, although dark vision is entirely lost^24^. This finding therefore represents a novel clinical phenotype caused by *SLC24A1* mutations.

**Figure 4:**
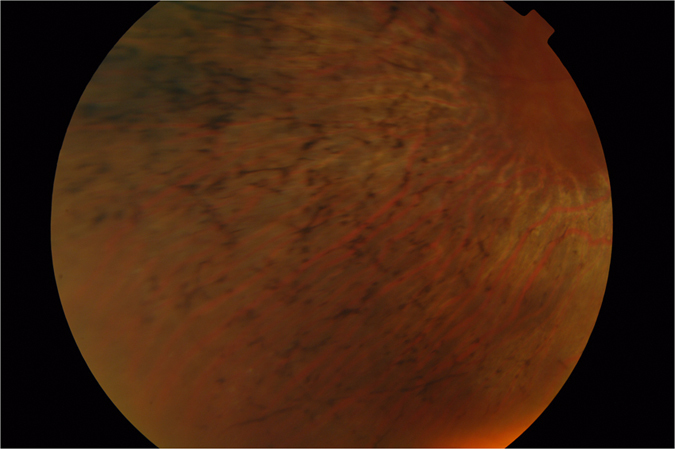
Fundus photograph of an affected patient from the SLC24A1-RP pedigree. Image quality is suboptimal because of the presence of cataracts, although pigmentary deposits, supporting the diagnosis of retinitis pigmentosa, are clearly visible.

In both the *GNAT1* and the *SLC24A1* pedigrees, the mutations were recessive premature-stop mutations, causing loss of protein function. In both pedigrees onset of the disease was late for RP, with a mild, slowly-progressing course. Similar to the GNAT1 phenotype^23^, patients in the *SLC24A1* pedigree presented with lifelong night-blindness, but with very distinctive, albeit mild, symptoms of RP including progressive mid-peripheral visual field loss, early appearance of cataracts and unmistakable pigmentary retinopathy on fundus photography (Fig. 4).

Notably, no other plausibly causative mutations were observed in either pedigree in the retinal genes included in the study. The data suggest that in both cases, a severe, homozygous mutation in a known CSNB disease gene has caused a mild, late-onset form of RP, and we therefore hypothesize that this may represent a general pattern in retinal disease. Additional observations of the phenotype resulting from severe mutations in CSNB-associated genes in other pedigrees will be required to confirm or refute this.

### Usher Syndrome

A total of 32 pedigrees with Usher syndrome were sequenced during the study, with type II disease being the most common (23 pedigrees), followed by type I (5 pedigrees). 4 other pedigrees had atypical or type III Usher syndrome. Consistent with previous work^25^,^26^, Usher Type I pedigrees were found to be predominantly (80%) caused by *MYO7A* mutations, while Usher Type II pedigrees were most commonly (74%) caused by mutations in *USH2A.* No causative mutations could be found in patients with atypical Usher syndrome, although two mutations were identified in the *CLRN1* gene in a patient with type III Usher syndrome.

### Choroideremia

Choroideremia is caused only by mutations in the *CHM* gene. As a result, detection was relatively uncomplicated and efficiencies were good. A total of 12 pedigrees with choroideremia were included in the study. Detection rates were the highest of all conditions included in this study; only two of the twelve pedigrees could not be solved. Three pedigrees in this cohort are particularly noteworthy: In one, the mutation NM_000390.2:c.1376C > G,p.Thr459Arg in the *CHM* gene was observed in all three affected members and no unaffected members, with no other relevant mutations being detected in that gene. This mutation is predicted by Human Splicing Finder^27^ to affect correct splicing and has not previously been reported as causative, and so this finding is of clinical relevance for future choroideremia diagnoses. In a second pedigree, a novel 6.3 kb deletion (hg19 co-ordinates chrX:85233437-85239772) was found to remove exons 3 and 4 of the *CHM* gene.

**Figure 5:**
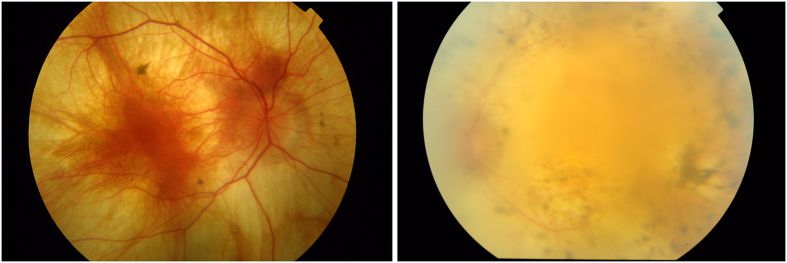
Left: Fundus photograph of a patient from the RPE65/CHM pedigree carrying a CHM mutation shows a classic choroideremia phenotype of optic disc pallor, mild vascular attenuation and relative preservation of the macula. Right: Fundus photograph of the patient from the RPE65/CHM pedigree carrying the homozygous RPE65 mutation shows some differences, including some bone-spicule deposits and poorer preservation of the macula.

A third pedigree, however, was an unusual case. NGS of the proband revealed a deleterious mutation in the *CHM* gene: NM_000390.2 c.715C > T,p.Arg239*, which was confirmed by Sanger sequencing. The pedigree structure was consistent with X-linked inheritance, with no affected females. A second affected member of the pedigree was Sanger sequenced, but was found not to carry the *CHM* mutation. At the time of assessment, this patient had an end-stage retinopathy which could not be cleanly classified, although it was presumed before sequencing was performed that they carried the same mutation and condition as the rest of the pedigree (see Fig. 5). The absence of the mutation was confirmed again using a fresh sample from the patient, indicating that the *CHM* mutation was definitively not present. NGS of this patient revealed a homozygous mutation in the *RPE65* gene (NM_000329.2:c.271C > T,p.Arg91Trp), which is known to be associated with retinitis pigmentosa^28^. Subsequent clinical work confirmed a diagnosis of *RPE65*-RP, making this was a very unusual case of two rare, genetically distinct Mendelian retinal diseases segregating within the same pedigree.

## Discussion

Adopting a target panel-based NGS approach targeting the exons of known retinal disease genes, causative mutations could be identified in 57**%** of pedigrees (a breakdown by condition is given in Fig. 2). The fraction of pedigrees for which causative mutations could be identified varied widely between retinal conditions, with conditions such as Stargardt disease and choroideremia having the highest detection rates (70–86%).

A major novel finding from this study was the association of two genes, *GNAT1* and *SLC24A1*, with RP that had previously only been associated with congenital stationary night blindness (CSNB). Although both retinitis pigmentosa and stationary night blindness affect rod photoreceptor cells, they are considered to be distinct conditions. In both cases, the mutation was a complete loss of function, either a homozygous premature stop or a homozygous frameshift. This suggests the intriguing possibility of a recurring pattern: Severe variants in CSNB-associated genes can cause a mild form of RP, potentially blurring the distinction between CSNB- and RP -associated genes, although the data is too limited for strong conclusions to be drawn as yet.

The results of this study emphasize the diversity of mutations underlying IRDs in the Irish population and the significant value of targeted NGS for IRDs. More than forty novel, previously unreported mutations were identified in this patient cohort (Table 1). We demonstrate the application of novel ensemble prediction methods and protein folding simulations for variant effect prediction (Fig. 3). In other cases, mutations in unexpected genes, such as BBS1 mutations in cases of nonsyndromic RP, underscored the significant overlap between different conditions in terms of clinical presentations, as well as the difficulty in identifying the causative gene based on clinical examination alone. The observation of one pedigree in which two IRDs are segregating (*RPE65*-RP and *CHM*-Choroideremia) serves to emphasise the essential role that NGS will play in the future diagnosis of this genetically heterogeneous group of conditions.

The pedigrees for which a mutation could not be identified are likely to be a mixture of those which could not be observed, such as mutations in promoter regions and other non-coding regions or genes not included in the panel, and those where the true mutations were in the sequenced region but could not be uniquely identified as causative because of the presence of multiple candidates. It is difficult to assess what proportion of pedigrees fall into each category, however an estimate can be made given that just over 25% of pedigrees had no candidate variants remaining in relevant genes after common and synonymous mutations were filtered out of the analysis. We suggest that this may be a reasonable, albeit conservative, lower bound for the fraction of pedigrees that may have non-coding or novel-gene mutations requiring whole-exome or whole-genome sequencing.

This issue informs the question of whether to select whole-genome or targeted-panel sequencing for future studies. Costs of NGS can be broken down between library preparation and sequencing. Library preparation for a targeted panel of genes is more expensive in terms of time and reagents than preparing a whole-genome library, however, this is outweighed by the cost of deep-coverage whole-genome sequencing, which is still about $1000/sample, 10 or 20 times the cost for sequencing of pooled target-capture samples.

Until whole-genome sequencing costs fall to a half or a quarter of current levels, therefore, there is still a role for targeted sequencing due to lower cost and the simplicity of analysis. For the near-term future, we recommend a two-tier approach to genetic analysis of IRDs: targeted sequencing of a core panel of exons to identify mutations in known genes, followed by whole-exome or whole-genome sequencing for pedigrees in which a mutation could not be found by the targeted approach. This applies even when the aim of the study is to identify non-coding mutations and new disease genes, as the cost savings from eliminating 50–60% of patients with coding mutations in known genes from the larger-scale sequencing study would more than outweigh the cost of performing targeted sequencing. The combination of these approaches, in concert with the increasing range of known retinopathy genes and the decreasing cost of wider-scale sequencing approaches, is likely to improve mutation detection rates even further in the next few years.

## Methods

### Patient Identification and Recruitment

Probands and other family members were primarily assessed at the Research Foundation of the Royal Victoria Eye and Ear Hospital (Dublin, Ireland) and the Mater Misericordiae University Hospital (Dublin, Ireland). With informed consent, best-corrected visual acuity was assessed using revised 2000 ETDRS charts (Precision Vision, La Salle Il, USA). Color vision was examined using the Lanthony desaturated panel D-15^29^ under standardised lighting conditions. Goldmann perimetry was used to assess the peripheral visual fields to the IV4e, I4e and 04e targets. Full-field electroretinograms were performed according to ISCEV standards^30^ using a Roland Consult RETI-port retiscan (Brandenburg an der Havel, Germany). Fundus color and autofluorescence photography was performed using a Topcon CRC50DX. Spectral domain optical coherence tomography was performed using a Cirrus HD-OCT (Carl Zeiss Meditec, Germany).

### DNA Isolation and Sequencing

Following informed consent, blood samples were collected from patients after clinical assessment. DNA was isolated from 2 ml of patient blood and fragmented for sequencing by ultrasonication in a Diagenode Bioruptor (Diagenode s.a., Belgium) to an average fragment size of 200–250 bp.

Sequencing libraries were generated and target capture was performed initially using the Agilent Sureselect XT2 kit (Agilent Technologies, Santa Clara, CA). Later captures used a redesigned panel with the Roche Nimblegen SeqCap EZ kit (Roche), incorporating new genes implicated in retinopathies since the design of the earlier panel. For both kits, captures were performed according to the manufacturer’s recommendations. Exons for all genes previously implicated in retinal degeneration, as listed by Retnet at the time of capture panel design, were included as capture targets, plus 100 bp surrounding the CEP290 intronic mutation implicated in LCA^31^. Additional intronic regions in ABCA4^32^ and USH2A^33^ that are commonly implicated in disease were not included in the panel but were sequenced by single-read sequencing in relevant patients when causative mutations were not detected by targeted sequencing. These regions will be included in future versions of the panel. In the earlier capture design, UTRs were also included, but these were excluded in later panels to improve exon coverage and sequencing throughput. The total size of the captured region was 1,490 kb for the earlier captures and 728 kb for the later captures.

Captured patient DNA was multiplexed into either 24- or 96-sample pools and sent for sequencing. Sequencing of 96-sample pools was performed off-site by BGI Tech using an Illumina HiSeq 2000 (Illumina, San Diego, CA). 24-sample pools were sequenced locally using an Illumina MiSeq. Confirmatory single-read sequencing was also performed to verify the presence of candidate mutations.

### Data Analysis

Sequence data were demultiplexed and mapped to the human genome (hg19) using BWA version 0.7.12^34^. Duplicate reads were flagged using Picard version 1.106^35^ and downstream analysis and variant calling were performed using GATK version 3.3.0^36^ according to the protocol specified in the GATK Best Practices Workflow, with the notable difference that hard-filtering rather than variant quality score recalibration (VQSR) was used to filter variants, due to the small size of the capture area. Variants filtered by this method were not discarded, but instead marked as potential sequencing artefacts.

The list of identified variants was annotated with snpEFF^37^ and dbNSFP^38^, and an ensemble model was used for classification of novel variants. Commonly used variant effect prediction software is based on machine learning approaches that learn a classifier model based on patterns in datasets of known pathogenic and neutral mutations^39^,^40^. In the fields of statistics and machine learning, ‘ensemble’ classifier models are commonly used which learn meta-classifiers using the predictions of existing individual classifier models as inputs. Such models frequently have performances superior to any individual model in the ensemble^41^, particularly if, as is the case with variant effect prediction, the individual models employ different approaches, features and training sets to generate their models and therefore have errors that should be substantially decorrelated with each other. The primary tool adopted for novel variant pathogenicity prediction was therefore an ensemble prediction model^42^, which shows significant improvements in accuracy compared to many tools, but which to our knowledge has not yet been used in studies of IRDs.

Synonymous variants and common polymorphisms were filtered out, and the remaining list of rare variants with the potential to affect protein sequence was output for manual curation. The output for each patient also included a list of coding regions where coverage was insufficient for reliable variant calling.

### Ethical Approval

Ethical approval for this study was granted by the ethics committee of the Royal Victoria Eye and Ear Hospital prior to commencement of this study. All work was carried out in accordance with the approved guidelines. All patients gave written informed consent before recruitment to the study. No patients under 18 years of age were included in the study.

## Additional Information

**How to cite this article**: Carrigan, M. *et al*. Panel-Based Population Next-Generation Sequencing for Inherited Retinal Degenerations. *Sci. Rep.* **6**, 33248; doi: 10.1038/srep33248 (2016).

## Figures and Tables

**Table 1:** List of novel disease-associated mutations identified in this study.

Gene	Condition	Transcript	DNA change	Protein change	Notes
BBS1	Bardet-Biedl Syndrome	NM_024649.4	c.1514_1515delTG	p.Leu505fs	In conjunction with p.Met390Arg
BBS9	Bardet-Biedl Syndrome	NM_198428.2	c.542C > G	p.Pro181Arg	Homozygous
BEST1	Best Vitelliform Macular Dystrophy	NM_001139443.1	c.241C > A	p.Arg81Ser	In conjunction with p.Ile141Thr
CHM	Choroideremia	NM_000390.2	c.476dupC	p.Ser160fs	Hemizygous
CHM	Choroideremia	NM_000390.2	c.1164delC	p.Cys388fs	Hemizygous
CHM	Choroideremia	NM_000390.2	c.1376C > G	p.Thr459Arg	Hemizygous
CHM	Choroideremia	NM_000390.2	c.757C > T	p.Arg253*	Hemizygous
CHM	Choroideremia	NM_000390.2	c.808C > T	p.Arg270*	Hemizygous
CNGA3	Stationary Night Blindness (recessive)	NM_001298.2	c.1535A > T	p.Lys512Met	In conjunction with p.His36fs
CNGA3	Stationary Night Blindness (recessive)	NM_001298.2	c.107_110delACTC	p.His36fs	In conjunction with p.Lys512Met
IQCB1	Leber Congenital Amaurosis (recessive)	NM_001023570.2	c.1036G > T	p.Glu346*	In conjunction with p.Arg489*
IQCB1	Leber Congenital Amaurosis (recessive)	NM_001023570.2	c.1465C > T	p.Arg489*	In conjunction with p.Glu346*
LCA5	Leber Congenital Amaurosis (recessive)	NM_001122769.2	c.1756A > T	p.Lys586*	Homozygous
NR2E3	Goldmann-Favre	NM_016346.3	c.328dupC	p.Gln110fs	Homozygous
NRL	Atypical Leber Congenital Amaurosis (recessive)	NM_006177.3	c.16delA	p.Ser6fs	In conjunction with p.Ala129fs
NRL	Atypical Leber Congenital Amaurosis (recessive)	NM_006177.3	c.386delC	p.Ala129fs	In conjunction with p.Ser6fs
PRPF31	Retinitis Pigmentosa (dominant)	NM_015629.3	c.1190dupG	p.His398fs	Heterozygous
PRPF8	Retinitis Pigmentosa (dominant)	NM_006445.3	c.6930G > C	p.Arg2310Ser	Heterozygous
RHO	Retinitis Pigmentosa (dominant)	NM_000539.3	c.439C > T	p.Arg147Cys	Heterozygous
RHO	Retinitis Pigmentosa (dominant)	NM_000539.3	c.754dupC	p.Arg252fs	Heterozygous
RHO	Retinitis Pigmentosa (dominant)	NM_000539.3	c.541G > A	p.Glu181Lys	Heterozygous
RP1	Retinitis Pigmentosa (dominant)	NM_006269.1	c.2107_2108dupAA	p.Asn703fs	Heterozygous
RP1	Retinitis Pigmentosa (dominant)	NM_006269.1	c.2285_2289delTAAAT	p.Leu762fs	Heterozygous
RP1	Retinitis Pigmentosa (dominant)	NM_006269.1	c.4090A > T	p.Arg1364*	Heterozygous
RP1	Retinitis Pigmentosa (dominant)	NM_006269.1	c.2348dupA	p.Asn783fs	Heterozygous
RPGR	Retinitis Pigmentosa (X-linked)	NM_001034853.1	c.2405_2406delAG	p.Glu802fs	Hemizygous
RPGR	Retinitis Pigmentosa (X-linked)	NM_001034853.1	c.295A > G	p.Thr99Ala	Hemizygous
RPGR	Retinitis Pigmentosa (X-linked)	NM_001034853.1	c.1928C > G	p.Ser643*	Hemizygous
RPGR	Retinitis Pigmentosa (X-linked)	NM_001034853.1	c.2007G > A	p.Trp669*	Hemizygous
RS1	Retinoschisis (X-linked)	NM_000330.3	c.413C > A	p.Thr138Asn	Hemizygous
SDCCAG8	Bardet-Biedl Syndrome	NM_006642.3	c.696T > G	p.Tyr232*	In conjunction with p.Arg374*
SDCCAG8	Bardet-Biedl Syndrome	NM_006642.3	c.1120C > T	p.Arg374*	In conjunction with p.Tyr232*
SLC24A1	Retinitis Pigmentosa	NM_004727.2	c.2679delT	p.Asn893fs	Homozygous
USH2A	Usher Syndrome	NM_206933.2	c.3187_3188delCA	p.Gln1063fs	Homozygous
USH2A	Usher Syndrome	NM_206933.2	c.12819T > A	p.Tyr4273*	In conjunction with p.Val218Glu

Amino acid substitutions were subject to stringent evaluation before inclusion in the list and were only included if segregation with disease could be confirmed in at least three family members, including at least two affected individuals, and bioinformatic methods predicted the mutation to be damaging to protein function.
